# 4-Hy­droxy-3-meth­oxy­benzaldehyde 4-ethyl­thio­semicarbazone

**DOI:** 10.1107/S1600536814016018

**Published:** 2014-07-17

**Authors:** Adriano Bof de Oliveira, Johannes Beck, Jörg Daniels, Bárbara Regina Santos Feitosa

**Affiliations:** a Departamento de Química, Universidade Federal de Sergipe, Av. Marechal Rondon s/n, Campus, 49100-000 São Cristóvão–SE, Brazil; b Institut für Anorganische Chemie, Universität Bonn, Gerhard-Domagk-Strasse 1, D-53121 Bonn, Germany

**Keywords:** Synthesis thio­semicarbazones, biological properties of thio­semicarbazones., crystal structure

## Abstract

In the crystal structure of the title compound, C_11_H_15_N_3_O_2_S, the C—N—N—C and C—N—C—C torsion angles involving the benzene ring and ethyl group are 11.91 (15) and 99.4 (2)°, respectively. An intra­molecular N—H⋯N hydrogen bond is observed. In the crystal, mol­ecules are linked *via* N—H⋯O and N—H⋯S hydrogen bonds into a three-dimensional hydrogen bonded network. Finally, the molecules show a herringbone arrangement when viewed along the *a* axis.

## Related literature   

For the synthesis and biological applications of thio­semicarbazone derivatives, see: Lovejoy & Richardson (2008 ▶). For one of the first reports on the synthesis of thio­semicarbazone derivatives, see: Freund & Schander (1902 ▶).
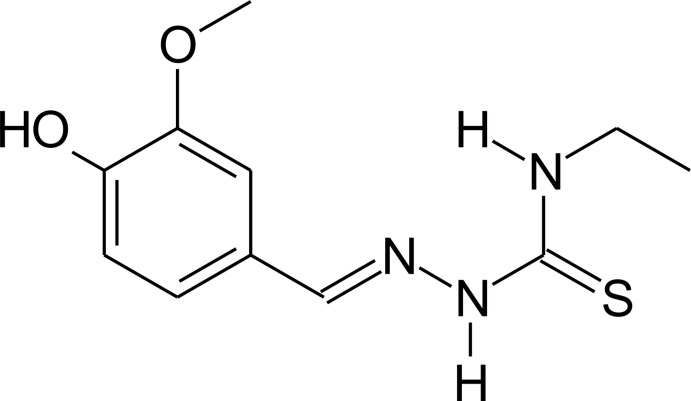


## Experimental   

### 

#### Crystal data  

C_11_H_15_N_3_O_2_S
*M* *_r_* = 253.32
Orthorhombic, 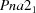

*a* = 8.9962 (2) Å
*b* = 16.1159 (2) Å
*c* = 8.5491 (1) Å
*V* = 1239.46 (3) Å^3^
*Z* = 4
Mo *K*α radiation
μ = 0.26 mm^−1^
*T* = 293 K
0.15 × 0.13 × 0.12 mm

#### Data collection  

Nonius Kappa CCD diffractometer
Absorption correction: multi-scan (Blessing, 1995 ▶) *T*_min_ = 0.939, *T*_max_ = 0.990
22619 measured reflections
2837 independent reflections
2590 reflections with *I* > 2σ(*I*)
*R*_int_ = 0.050

#### Refinement  

*R*[*F* ^2^ > 2σ(*F* ^2^)] = 0.030
*wR*(*F* ^2^) = 0.071
*S* = 1.01
2837 reflections
214 parameters
1 restraint
All H-atom parameters refined
Δρ_max_ = 0.15 e Å^−3^
Δρ_min_ = −0.23 e Å^−3^
Absolute structure: Flack (1983 ▶)
Absolute structure parameter: 0.03 (6)

### 

Data collection: *COLLECT* (Nonius, 1998 ▶); cell refinement: *HKL* *SCALEPACK* (Otwinowski & Minor, 1997 ▶); data reduction: *HKL*, *DENZO* (Otwinowski & Minor, 1997 ▶) and *SCALEPACK*; program(s) used to solve structure: *SHELXS97* (Sheldrick, 2008 ▶); program(s) used to refine structure: *SHELXL97* (Sheldrick, 2008 ▶); molecular graphics: *DIAMOND* (Brandenburg, 2006 ▶); software used to prepare material for publication: *publCIF* (Westrip, 2010 ▶).

## Supplementary Material

Crystal structure: contains datablock(s) I, publication_text. DOI: 10.1107/S1600536814016018/bx2462sup1.cif

Structure factors: contains datablock(s) I. DOI: 10.1107/S1600536814016018/bx2462Isup2.hkl

Supporting information file. DOI: 10.1107/S1600536814016018/bx2462Isup3.cml

CCDC reference: 1013029

Additional supporting information:  crystallographic information; 3D view; checkCIF report

## Figures and Tables

**Table 1:** Hydrogen-bond geometry (Å, °)

*D*—H⋯*A*	*D*—H	H⋯*A*	*D*⋯*A*	*D*—H⋯*A*
O2—H*O*2⋯S1^i^	0.86 (3)	2.26 (3)	3.1144 (14)	173 (2)
N3—H*N*3⋯N1	0.77 (2)	2.25 (2)	2.643 (2)	112.4 (19)
N3—H*N*3⋯O2^ii^	0.77 (2)	2.43 (2)	3.023 (2)	135 (2)
N3—H*N*3⋯O1^ii^	0.77 (2)	2.52 (2)	3.061 (2)	128.3 (19)

Symmetry codes: (i) 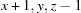; (ii) 
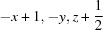
.

**Fig. 1.**
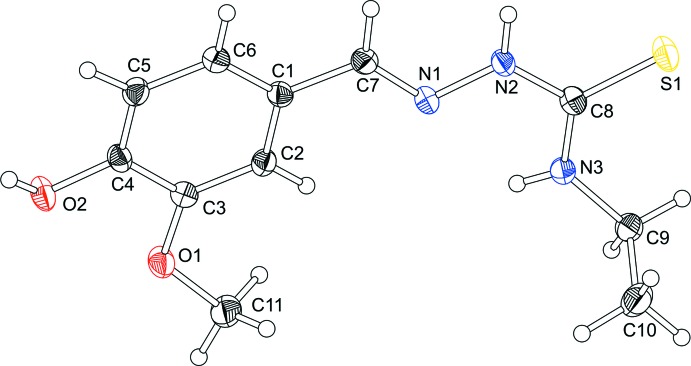
The molecular structure of the title compound with labeling and displacement ellipsoids drawn at the 50% probability level.

**Fig. 2.**
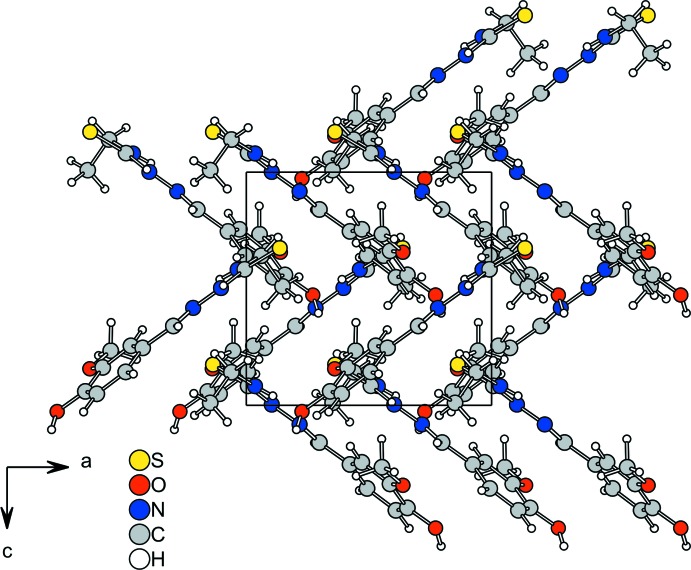
Crystal structure of the title compound viewed along the b-axis. The herringbone pattern of the crystal packing along the a-axis is observed.

## supplementary crystallographic information

### S1. Related Literature

For Biological activities of thio­semicarbazone derivatives see Lovejoy & Richardson, 2008.

### S2. Comment

Thiosemicarbazone derivatives have a wide range of biological properties. For example, some thiosemicarbazones show anti-proliferative activity against tumor cells (Lovejoy & Richardson, 2008). As part of our study on synthesis and structural chemistry of thiosemicarbazone derivatives from natural products, we report herein the crystal structure of a derivative of vanillin.

In the title compound, C_11_H_15_N_3_O_2_S, Fig. 1, the C-N-N-C and C–N–C–C fragments makes torsion angles of 11.91 (15)° and 99.4 (2)° with the benzene ring and ethyl group respectively. The molecule matches the asymmetric unit (Fig. 1) and shows a *trans* conformation at the C7—N1 and N1—N2 bonds. In the crystal structure the molecules are linked *via* N—H···O and O—H···S hydrogen bonds interactions into a crystal packing which shows a herringbone arrangement viewed along the *a*-axis,Fig.2. Additionally, one N—H···N intramolecular hydrogen bond interactions is observed, Table 1,

### S3. Experimental

Starting materials were commercially available and were used without further purification. The synthesis of the title compound was adapted to a procedure reported previously (Freund & Schander, 1902). In a hydrochloric acid catalyzed reaction, a mixture of vanillin (10 mmol) and 4-ethyl-3-thiosemicarbazide (10 mmol) in ethanol (80 ml), was refluxed for 5 h. After cooling and filtering, the title compound was obtained. Crystals suitable for X-ray diffraction were obtained in ethanol by the slow evaporation of solvent.

### S4. Refinement

All hydrogen atoms were localized in a difference density Fourier map. Their positions and isotropic displacement parameters were refined.

### Crystal data

**Table:**

C_11_H_15_N_3_O_2_S	*F*(000) = 536
*M**_r_* = 253.32	*D*_x_ = 1.358 Mg m^−^^3^
Orthorhombic, *P**n**a*2_1_	Mo *K*α radiation, λ = 0.71073 Å
Hall symbol: P 2c -2n	Cell parameters from 31793 reflections
*a* = 8.9962 (2) Å	θ = 2.9–27.5°
*b* = 16.1159 (2) Å	µ = 0.26 mm^−^^1^
*c* = 8.5491 (1) Å	*T* = 293 K
*V* = 1239.46 (3) Å^3^	Prism, yellow
*Z* = 4	0.15 × 0.13 × 0.12 mm

### Data collection

**Table:**

Nonius Kappa CCD diffractometer	2837 independent reflections
Radiation source: fine-focus sealed tube, Nonius KappaCCD	2590 reflections with *I* > 2σ(*I*)
Graphite monochromator	*R*_int_ = 0.050
Detector resolution: 9 pixels mm^-1^	θ_max_ = 27.5°, θ_min_ = 3.4°
CCD rotation images, thick slices scans	*h* = −11→11
Absorption correction: multi-scan (Blessing, 1995)	*k* = −20→20
*T*_min_ = 0.939, *T*_max_ = 0.990	*l* = −11→11
22619 measured reflections	

### Refinement

**Table:**

Refinement on *F*^2^	Secondary atom site location: difference Fourier map
Least-squares matrix: full	Hydrogen site location: inferred from neighbouring sites
*R*[*F*^2^ > 2σ(*F*^2^)] = 0.030	All H-atom parameters refined
*wR*(*F*^2^) = 0.071	*w* = 1/[σ^2^(*F*_o_^2^) + (0.0355*P*)^2^ + 0.3575*P*] where *P* = (*F*_o_^2^ + 2*F*_c_^2^)/3
*S* = 1.01	(Δ/σ)_max_ < 0.001
2837 reflections	Δρ_max_ = 0.15 e Å^−^^3^
214 parameters	Δρ_min_ = −0.23 e Å^−^^3^
1 restraint	Absolute structure: Flack (1983), ???? Friedel pairs
Primary atom site location: structure-invariant direct methods	Absolute structure parameter: 0.03 (6)

### Special details

**Table:**

Geometry. All e.s.d.'s (except the e.s.d. in the dihedral angle between two l.s. planes) are estimated using the full covariance matrix. The cell e.s.d.'s are taken into account individually in the estimation of e.s.d.'s in distances, angles and torsion angles; correlations between e.s.d.'s in cell parameters are only used when they are defined by crystal symmetry. An approximate (isotropic) treatment of cell e.s.d.'s is used for estimating e.s.d.'s involving l.s. planes.
Refinement. Refinement of *F*^2^ against ALL reflections. The weighted *R*-factor *wR* and goodness of fit *S* are based on *F*^2^, conventional *R*-factors *R* are based on *F*, with *F* set to zero for negative *F*^2^. The threshold expression of *F*^2^ > σ(*F*^2^) is used only for calculating *R*-factors(gt) *etc*. and is not relevant to the choice of reflections for refinement. *R*-factors based on *F*^2^ are statistically about twice as large as those based on *F*, and *R*- factors based on ALL data will be even larger.

### Fractional atomic coordinates and isotropic or equivalent isotropic displacement parameters (Å^2^)

**Table:**

	*x*	*y*	*z*	*U*_iso_*/*U*_eq_	
S1	−0.13718 (5)	−0.18485 (2)	−0.32559 (6)	0.02743 (12)	
O1	0.63909 (13)	0.01577 (7)	−0.84398 (16)	0.0256 (3)	
O2	0.77231 (14)	−0.08623 (8)	−1.02714 (15)	0.0254 (3)	
N1	0.21760 (16)	−0.15200 (8)	−0.58307 (17)	0.0196 (3)	
N2	0.10393 (16)	−0.18657 (9)	−0.49707 (18)	0.0212 (3)	
N3	0.03634 (18)	−0.05684 (9)	−0.41867 (19)	0.0210 (3)	
C1	0.42396 (18)	−0.17397 (10)	−0.7544 (2)	0.0189 (3)	
C2	0.47154 (19)	−0.09078 (10)	−0.7448 (2)	0.0190 (3)	
C3	0.58669 (18)	−0.06350 (9)	−0.8387 (2)	0.0195 (3)	
C4	0.65784 (18)	−0.11894 (11)	−0.9413 (2)	0.0193 (3)	
C5	0.61052 (19)	−0.20035 (11)	−0.9515 (2)	0.0211 (3)	
C6	0.49352 (18)	−0.22780 (10)	−0.8580 (2)	0.0208 (3)	
C7	0.29879 (19)	−0.20341 (11)	−0.6600 (2)	0.0199 (3)	
C8	0.00811 (18)	−0.13729 (10)	−0.4166 (2)	0.0194 (3)	
C9	−0.0612 (2)	0.00841 (10)	−0.3584 (2)	0.0242 (4)	
C10	−0.1481 (2)	0.04899 (14)	−0.4896 (2)	0.0328 (4)	
C11	0.5543 (2)	0.07826 (11)	−0.7660 (3)	0.0306 (4)	
HO2	0.795 (3)	−0.1171 (16)	−1.105 (3)	0.052 (8)*	
HN2	0.094 (2)	−0.2387 (13)	−0.487 (2)	0.019 (5)*	
HN3	0.109 (2)	−0.0442 (13)	−0.461 (2)	0.022 (5)*	
H2	0.425 (2)	−0.0549 (12)	−0.676 (2)	0.022 (5)*	
H5	0.664 (2)	−0.2354 (12)	−1.029 (2)	0.021 (5)*	
H6	0.461 (2)	−0.2851 (12)	−0.866 (2)	0.026 (5)*	
H7	0.2793 (19)	−0.2637 (12)	−0.662 (2)	0.017 (4)*	
H9A	0.008 (2)	0.0526 (12)	−0.307 (2)	0.023 (5)*	
H9B	−0.129 (2)	−0.0134 (12)	−0.279 (2)	0.022 (5)*	
H10A	−0.078 (3)	0.0751 (15)	−0.573 (3)	0.047 (7)*	
H10B	−0.212 (2)	0.0056 (12)	−0.544 (3)	0.028 (5)*	
H10C	−0.215 (3)	0.0921 (15)	−0.450 (3)	0.046 (6)*	
H11A	0.604 (2)	0.1297 (13)	−0.788 (3)	0.032 (6)*	
H11B	0.448 (3)	0.0771 (13)	−0.803 (3)	0.038 (6)*	
H11C	0.553 (3)	0.0652 (14)	−0.645 (3)	0.045 (7)*	

### Atomic displacement parameters (Å^2^)

**Table:**

	*U*^11^	*U*^22^	*U*^33^	*U*^12^	*U*^13^	*U*^23^
S1	0.0304 (2)	0.02365 (19)	0.0283 (2)	−0.00829 (17)	0.0134 (2)	−0.0068 (2)
O1	0.0267 (6)	0.0195 (5)	0.0305 (7)	−0.0043 (5)	0.0100 (6)	−0.0047 (6)
O2	0.0264 (6)	0.0264 (6)	0.0234 (7)	−0.0068 (5)	0.0098 (5)	−0.0045 (5)
N1	0.0187 (7)	0.0211 (7)	0.0190 (7)	−0.0021 (6)	0.0033 (6)	0.0017 (6)
N2	0.0221 (7)	0.0163 (7)	0.0253 (8)	−0.0019 (6)	0.0089 (6)	0.0004 (6)
N3	0.0207 (7)	0.0180 (7)	0.0244 (8)	−0.0002 (6)	0.0054 (6)	0.0001 (6)
C1	0.0183 (8)	0.0217 (8)	0.0168 (7)	0.0011 (6)	−0.0002 (7)	0.0038 (6)
C2	0.0181 (8)	0.0205 (8)	0.0182 (8)	0.0023 (6)	0.0014 (7)	−0.0012 (7)
C3	0.0207 (7)	0.0184 (7)	0.0192 (8)	−0.0006 (6)	−0.0001 (7)	0.0011 (7)
C4	0.0191 (8)	0.0235 (8)	0.0154 (7)	−0.0009 (6)	0.0022 (7)	0.0021 (6)
C5	0.0217 (8)	0.0217 (8)	0.0200 (9)	0.0020 (6)	0.0019 (7)	−0.0016 (7)
C6	0.0209 (8)	0.0188 (7)	0.0226 (8)	−0.0005 (6)	0.0008 (7)	0.0013 (7)
C7	0.0212 (8)	0.0202 (8)	0.0182 (8)	0.0006 (7)	0.0006 (7)	0.0013 (7)
C8	0.0208 (8)	0.0213 (8)	0.0162 (7)	−0.0021 (7)	0.0002 (7)	−0.0015 (7)
C9	0.0299 (9)	0.0190 (7)	0.0238 (9)	0.0026 (7)	0.0081 (8)	−0.0024 (7)
C10	0.0321 (10)	0.0347 (10)	0.0316 (10)	0.0121 (9)	0.0042 (9)	−0.0003 (9)
C11	0.0323 (11)	0.0192 (9)	0.0404 (12)	−0.0005 (8)	0.0100 (9)	−0.0054 (8)

### Geometric parameters (Å, º)

**Table:**

S1—C8	1.7035 (17)	C2—H2	0.93 (2)
O1—C3	1.3625 (18)	C3—C4	1.406 (2)
O1—C11	1.429 (2)	C4—C5	1.382 (2)
O2—C4	1.370 (2)	C5—C6	1.394 (2)
O2—HO2	0.86 (3)	C5—H5	0.99 (2)
N1—C7	1.286 (2)	C6—H6	0.97 (2)
N1—N2	1.377 (2)	C7—N1	1.286 (2)
N2—C8	1.359 (2)	C7—H7	0.988 (18)
N2—N1	1.377 (2)	C9—C10	1.515 (3)
N2—HN2	0.85 (2)	C9—H9A	1.046 (19)
N3—C8	1.321 (2)	C9—H9B	0.98 (2)
N3—C9	1.463 (2)	C10—H10A	1.04 (3)
N3—HN3	0.77 (2)	C10—H10B	1.02 (2)
C1—C6	1.389 (2)	C10—H10C	0.98 (3)
C1—C2	1.410 (2)	C11—H11A	0.96 (2)
C1—C7	1.464 (2)	C11—H11B	1.00 (2)
C2—C3	1.382 (2)	C11—H11C	1.06 (3)
			
C3—O1—C11	117.43 (14)	C5—C6—H6	119.1 (12)
C4—O2—HO2	112.0 (18)	N1—C7—C1	120.67 (15)
C7—N1—N2	115.75 (14)	N1—C7—C1	120.67 (15)
C8—N2—N1	120.31 (14)	N1—C7—H7	122.7 (11)
C8—N2—N1	120.31 (14)	N1—C7—H7	122.7 (11)
C8—N2—HN2	117.5 (13)	C1—C7—H7	116.6 (11)
N1—N2—HN2	122.1 (13)	N3—C8—N2	116.42 (15)
N1—N2—HN2	122.1 (13)	N3—C8—S1	126.51 (13)
C8—N3—C9	125.82 (15)	N2—C8—S1	117.07 (12)
C8—N3—HN3	115.3 (16)	N3—C9—C10	111.04 (15)
C9—N3—HN3	118.8 (16)	N3—C9—H9A	106.1 (10)
C6—C1—C2	119.64 (15)	C10—C9—H9A	109.0 (11)
C6—C1—C7	119.69 (15)	N3—C9—H9B	111.2 (11)
C2—C1—C7	120.63 (15)	C10—C9—H9B	110.1 (11)
C3—C2—C1	119.74 (15)	H9A—C9—H9B	109.3 (16)
C3—C2—H2	120.7 (12)	C9—C10—H10A	111.8 (14)
C1—C2—H2	119.5 (12)	C9—C10—H10B	109.3 (12)
O1—C3—C2	125.22 (15)	H10A—C10—H10B	108.0 (18)
O1—C3—C4	114.69 (14)	C9—C10—H10C	111.4 (15)
C2—C3—C4	120.09 (14)	H10A—C10—H10C	109 (2)
O2—C4—C5	124.25 (15)	H10B—C10—H10C	107.4 (17)
O2—C4—C3	115.59 (14)	O1—C11—H11A	105.6 (12)
C5—C4—C3	120.17 (15)	O1—C11—H11B	110.1 (13)
C4—C5—C6	119.85 (16)	H11A—C11—H11B	113.4 (17)
C4—C5—H5	115.7 (11)	O1—C11—H11C	108.9 (13)
C6—C5—H5	124.4 (11)	H11A—C11—H11C	111.5 (19)
C1—C6—C5	120.50 (15)	H11B—C11—H11C	107 (2)
C1—C6—H6	120.4 (12)		
			
C7—N1—N2—C8	−177.34 (16)	C7—C1—C6—C5	178.46 (16)
C6—C1—C2—C3	0.1 (3)	C4—C5—C6—C1	0.0 (3)
C7—C1—C2—C3	−177.84 (15)	N2—N1—C7—C1	−179.36 (15)
C11—O1—C3—C2	−10.6 (3)	C6—C1—C7—N1	−168.55 (16)
C11—O1—C3—C4	168.32 (16)	C2—C1—C7—N1	9.4 (3)
C1—C2—C3—O1	177.64 (16)	C6—C1—C7—N1	−168.55 (16)
C1—C2—C3—C4	−1.2 (3)	C2—C1—C7—N1	9.4 (3)
O1—C3—C4—O2	2.2 (2)	C9—N3—C8—N2	171.47 (17)
C2—C3—C4—O2	−178.86 (14)	C9—N3—C8—S1	−7.6 (3)
O1—C3—C4—C5	−177.30 (15)	N1—N2—C8—N3	−4.0 (2)
C2—C3—C4—C5	1.6 (3)	N1—N2—C8—N3	−4.0 (2)
O2—C4—C5—C6	179.51 (15)	N1—N2—C8—S1	175.21 (13)
C3—C4—C5—C6	−1.0 (3)	N1—N2—C8—S1	175.21 (13)
C2—C1—C6—C5	0.5 (2)	C8—N3—C9—C10	−99.4 (2)

### Hydrogen-bond geometry (Å, º)

**Table:**

*D*—H···*A*	*D*—H	H···*A*	*D*···*A*	*D*—H···*A*
O2—H*O*2···S1^i^	0.86 (3)	2.26 (3)	3.1144 (14)	173 (2)
N3—H*N*3···N1	0.77 (2)	2.25 (2)	2.643 (2)	112.4 (19)
N3—H*N*3···O2^ii^	0.77 (2)	2.43 (2)	3.023 (2)	135 (2)
N3—H*N*3···O1^ii^	0.77 (2)	2.52 (2)	3.061 (2)	128.3 (19)

Symmetry codes: (i) *x*+1, *y*, *z*−1; (ii) −*x*+1, −*y*, *z*+1/2.
